# Safety and Tolerability of BRAF Inhibitor and BRAF Inhibitor-Based Combination Therapy in Chinese Patients With Advanced Melanoma: A Real World Study

**DOI:** 10.3389/fonc.2021.582676

**Published:** 2021-04-01

**Authors:** Xing Liu, Jing-jing Li, Ya Ding, Dan-dan Li, Xi-zhi Wen, De-sheng Weng, Jiu-hong Wang, Hang Jiang, Xiao-shi Zhang

**Affiliations:** Biotherapy Center, Sun Yat-sen University Cancer Center, Guangzhou, China

**Keywords:** BRAFV600E/K-positive, advanced melanoma, BRAF inhibitor, BRAF inhibitor-based combination, China

## Abstract

The toxicity spectrum between Chinese and Caucasian patients with melanoma who were treated with BRAF inhibitors (BRAFi) may differ. The purpose of the present study was to assess the safety and tolerability of BRAFi and BRAFi-based combination therapies [MEK inhibitors (MEKi) or anti-programmed death-1 (PD-1) antibody] in Chinese patients with *BRAF V600E/K* mutation-positive metastatic melanoma. We also investigated whether treatment-related adverse events (AEs) correlated with the prognosis. This retrospective study collected data from 43 patients with *BRAF V600E/K* mutation-positive metastatic melanoma from a single Chinese cancer center. Of the 43 patients, 12 patients received BRAFi monotherapy, 12 patients received BRAFi+MEKi, and 19 patients received BRAFi combined with the anti-PD-1 antibody. The median follow-up time was 19 months. In the BRAFi group, the most common AEs were rashes, palmoplantar erythrodysesthesia, and arthralgia. Four out of 12 (30%) patients experienced grade 3–4 treatment-related AEs. All grades of AEs in the BRAFi+MEKi group were similar to the BRAFi group, except for higher pyrexia (58.3%) and fewer cutaneous AEs. Three out of 12 (25%) patients experienced grade 3–4 AEs, especially pyrexia (16.7%). In the BRAFi+anti-PD-1 antibody group, AEs were similar to the BRAFi group, except for an increased aminotransferase level (36.8%), increased bilirubin (31.6%), and hypothyroidism (15.8%). Eleven out of 19 (57.9%) patients experienced grade 3–4 AEs and four out of 19 (21%) patients discontinued the therapy due to AEs. Treatment-related hepatotoxicity (trHE), defined as an increase in either alanine aminotransferase (ALT), aspartate transaminase (AST), or bilirubin levels, was the only AE identified as a significant poor-prognosis indicator in this study. The median progression-free survival of patients with trHE (41.9%) was 8 months, whereas it was 18 months for those without trHE [*p* = 0.046, hazard ratio (HR) = 2.116]. Moreover, this association was independent of medication regimens (*p* = 0.014, HR = 2.971). The overall response rate of patients with trHE was significantly lower than those without trHE (44.4 vs. 60.0%, *p* = 0.024), and we observed a similar trend in patients treated with BRAFi, BRAFi+MEKi, and BRAFi+anti-PD-1 antibody. In conclusion, BRAFi and BRAFi-based combination therapies were tolerable with reversible AEs in Chinese patients with melanoma. The trHE in patients receiving BRAFi and BRAFi-based regimens might indicate a poor therapy-related prognosis.

## Introduction

Melanoma is one of the most deadly diseases in China, with an estimated 5-year overall survival (OS) of merely 4.6% (1). *BRAF(V600E/K)* mutation, a component of the mitogen-activated protein kinase (MAPK) pathway, is regarded as a significant oncogene in melanoma. The overall response rate (ORR) of BRAF inhibitor monotherapy has been reported to be as high as 36–53% from clinical trials in Caucasian patients, with a median duration of response of merely 6–8 months (2–4). At present, patients with *BRAF V600*-mutant metastatic melanoma are recommended for combination treatment with BRAF inhibitors (BRAFi) and MEK inhibitors (MEKi), such as dabrafenib+trametinib (D+T), vemurafenib+cobimetinib (V+C), and encorafenib+binimetinib (E+B), because the combination can block the negative feedback loops for the activation of the MAPK pathway and delay the development of drug resistance (5–7).

Clinical characteristics, such as pathology, anatomical origin, and prognoses, differ significantly among different ethnic groups (8). The incidence of cutaneous melanoma is lower in Asian patients than in Caucasians (50–70% vs. 91.2%) (9, 10). Acral cutaneous melanoma has a higher incidence, accounting for up to 58% of all cutaneous melanomas in Asians, compared to Caucasians (1–7%) (11). Furthermore, Guo et al. reported that *BRAF* mutations in Chinese patients with melanoma were more frequent in non-acral cutaneous melanoma (43.3%) than in acral cutaneous melanoma; however, the frequencies reported by Maldona and Cohe in Caucasian non-acral cutaneous melanoma (60%) were still higher (12–14). Only one small study (*n* = 46) reported the toxicity spectrum of vemurafenib in Chinese patients with melanoma. By comparing data from this study with those from the pivotal BRIM-3 study (15), we found that the toxicity spectrum between Chinese and Caucasian patients with melanoma treated with vemurafenib was different. Chinese patients had a higher incidence of higher blood cholesterol levels (59 vs. <1%), hypertriglyceridemia (22 vs. <1%), total bile acid increase (22 vs. 0%), hyperuricemia (17 vs. <1%), serum bilirubin level increase (54 vs. 9%), leukopenia (22 vs. 0%), proteinuria (24 vs. <1%), and melanocytic nevus (52 vs. 10%). These differences may impact the completion of treatment. For example, an AE of grade 3, corresponding to an increase in serum cholesterol levels, led to an interruption in the treatment of Chinese patients with melanoma (15, 16). In addition, the safety and tolerability of the BRAFi+MEKi combination in Chinese patients with melanoma have not been reported. Therefore, the available data regarding the tolerability and safety of BRAFi and BRAFi+MEKi in Chinese patients are significant, especially from a real-world experience.

Recently, the combination of targeted therapy with immunotherapy was proposed to improve the long-term outcomes of patients. A preclinical study showed that BRAF/MEK-targeted therapies had effects, such as enhancing intratumor T-cell infiltration, increasing tumor antigens, and increasing the expression of programmed death-1 (PD-L1), on the tumor microenvironment, which supported their combination with PD-1/PD-L1 inhibitors (17–19). Ascierto et al. performed a randomized phase 2 trial enrolling 60 Caucasian patients with *BRAF V600*-mutant metastatic melanoma who were treated with a triple-combination therapy, including dabrafenib, trametinib, and pembrolizumab. Although progression-free survival (PFS) of the triple-combination was promising, grade 3–5 adverse events (AEs) occurred in 58.3% of patients and led to treatment discontinuation in 25 patients (41.7%) (20). A similar phenomenon could be observed in two other triple-treatment combinations (21, 22). However, the clinical experience relative to the tolerance of BRAFi, combined with immune-checkpoint inhibitors (ICIs), is still lacking in Chinese patients.

Thus, the purpose of this study was to analyze the safety and tolerability of BRAFi and BRAFi-based combination therapy (MEKi or anti-PD-1 antibody) in Chinese patients with *BRAF V600E/K* mutation-positive metastatic melanoma. Furthermore, we also investigated which treatment-related AE could represent as a predictor of efficacy.

## Materials and Methods

### Patients

A retrospective study of 43 previously treated or untreated advanced melanoma patients attending the Sun Yat-sen University Cancer Center between May 2015 and March 2020 was carried out. All data were extracted from the database containing electronic medical records of the institution. Patients were diagnosed with biopsy-confirmed advanced melanoma, and molecular profiling confirmed the presence of the *BRAF V600E/K* mutation. Only patients with a *BRAF* mutation who were treated with BRAFi (vemurafenib 960 mg, orally twice daily), BRAFi+MEKi [dabrafenib (D) 300 mg orally twice daily; trametinib (T) 2 mg orally once daily], or BRAFi+anti-PD-1 antibody [vemurafenib 960 mg orally twice daily, for 4–6 weeks, combined with pembrolizumab (2 mg/kg, every 3 weeks) once the disease was controlled) were eligible for analysis. Demographic, clinical, and survival data were retrieved from the medical records. All patients who accepted systemic therapy had a performance status of 0–2.

### Data Collection and Analysis

The following baseline characteristics were recorded for each patient: age, sex, Eastern Cooperative Oncology Group performance status (PS), disease stage (AJCC 8th edition), the number of disease sites, lactate dehydrogenase (LDH) levels, and the therapeutic regimen. AEs caused by the therapy were defined and graded according to the Common Terminology Criteria for Adverse Events, version 4.

Only *BRAF V600E/K* mutated patients receiving BRAFi, BRAFi+MEKi, or vemurafenib+pembrolizumab were enrolled in this study. The clinical data of all patients enrolled in this study were retrospectively analyzed. Efficacy included the ORR according to RECIST v1.1 criteria and was confirmed by repeat assessment at least 4 weeks after the criteria were first met. Tumor assessments [computed tomography (CT) or magnetic resonance imaging (MRI)] were obtained at screening, after 8–12 weeks (or as clinically indicated), until documented disease progression. PFS was estimated using Kaplan-Meier Statistical analysis. Safety assessments consisted of monitoring and recording of AEs. AEs were graded according to National Cancer Institute Common Terminology Criteria for Adverse Events, version 4.0.

### Statistical Analysis

Analysis of the correlation between AEs and PFS was performed. The median follow-up time was analyzed by the Reverse Kaplan-Meier method. Single continuous variables and categorical variables were examined with the Student's *t*-test and the chi-square tests, respectively. The multivariate Cox regression analysis was used to compare the PFS between patients who developed trHE and those who did not, and the analysis was adjusted for the baseline LDH level, medical regimens, and liver metastases. Statistical analyses were performed using the statistical packages SPSS (SPSS for Windows, version 22.0, SPSS Inc., Chicago, IL). All tests were two-tailed. Statistical significance was determined by a *p*-value < 0.05.

## Results

### Cohort Characteristics

In this study, 43 metastatic melanoma patients were eligible for analysis. Patients were treated with BRAFi (*n* = 12), BRAFi+MEKi (*n* = 12), and BRAFi+anti-PD-1 antibody (*n* = 19) as follows: vemurafenib (*n* = 12), D+T (*n* = 11) and V+C (*n* = 1), and vemurafenib+anti-PD-1 antibody (pembrolizumab) (*n* = 19). All patients had either a *BRAF V600E* mutation or a *BRAF V600K* mutation.

Baseline demographics and disease characteristics are shown in Table 1. Although baseline characteristics of the three groups were essentially the same, patients treated with vemurafenib combined with the anti-PD-1 antibody presented the best baseline values, including the lines of therapy, disease sites, and the LDH level. Most patients started the three regimens in this study as first or second-line (85.9%) therapy, and over half (57.9%) of the patients were treated with BRAFi+anti-PD-1 antibody as the first-line therapy. All patients had normal blood levels of aminotransferases [alanine aminotransferase (ALT) and aspartate transaminase (AST)] and bilirubin before treatment; however, 17 patients experienced hepatic metastasis and one patient was identified as positive for hepatitis B-antigen.

**Table 1:** Clinical characteristics of the study population (*n* = 43).

	**BRAFi**	**BRAFi+MEKi**	**BRAFi+PD-1 antibody**	**Total**
	***N* = 12**	***N* = 12**	***N* = 19**	***N* = 43**
**Sex—no. (%)**				
Male	5 (41.7)	6 (50.0)	13 (68.4)	24 (55.8)
Female	7 (58.3)	6 (50.0)	6 (31.6)	19 (44.2)
Median (range) age—years	52 (29–55)	44 (27–63)	49 (33–67)	47 (27–67)
**Performance status—no. (%)**				
0	1 (8.3)	9 (75.0)	13 (68.4)	23 (53.5)
1	10 (83.3)	2 (16.7)	6 (31.6)	18 (41.9)
2	1 (8.3)	1 (8.3)	0	2 (4.6)
**LDH—no. (%)**				
<ULN	7 (58.3)	9	14 (73.7)	32 (74.4)
>ULN	5 (41.7)	3 (25.0)	5 (26.3)	11 (25.6)
**Disease stage (AJCC#7)—no. (%)**				
M1a	2 (16.7)	3 (25.0)	9 (47.4)	14 (32.6)
M1b	3 (25.0)	4 (33.3)	5 (26.3)	12 (27.9)
M1c	6 (50.0)	4 (33.3)	2 (10.5)	12 (27.9)
M1d	1 (8.3)	1 (8.3)	3 (15.8)	5 (11.6)
**Disease site—no. (%)**				
≤3	4 (33.3)	7 (58.3)	12 (63.2)	23 (53.5)
>3	8 (66.7)	5 (41.7)	7 (36.8)	20 (46.5)
**Brain metastases—no. (%)**				
Yes	1 (8.3)	1 (8.3)	3 (15.8)	5 (11.7)
No	11 (91.7)	11 (91.7)	16 (84.2)	38 (88.3)
**Regime—no. (%)**				
Vemurafenib	12 (100.0)	-	-	12 (27.9)
Dabrafenib+trametinib	-	11 (91.7)	-	11 (25.6)
Vemurafenib+cobimetinib	-	1 (8.3)	-	1 (2.3)
Vemurafenib+pembrolizumab	-	-	19 (100)	19 (44.2)
**Basic liver function—no. (%)**				
Liver metastasis	6 (50.0)	5 (41.7)	5 (26.3)	16 (37.2)
Hepatitis B-antigen–positive.	0	0	1 (5.3)	1 (2.3)
Normal	6 (50.0)	7 (58.3)	13 (68.4)	26 (60.5)
**Line of therapy—no. (%)**				
1	4 (33.3)	4 (33.3)	11 (57.9)	19 (44.2)
2	6 (50.0)	6 (50.0)	7 (36.8)	19 (44.2)
3	2 (16.7)	1 (8.3)	1 (5.3)	4 (9.3)
4	0	1 (8.3)	0	1 (2.3)

*LDH, lactate dehydrogenase; ULN, upper normal limit; BRAFi, BRAF inhibitor; MEKi, MEK inhibitor*.

### Toxicity Profile of the Three Regimens

The total median follow-up time was 19 months (range, 11–26 months). At the time of data cutoff, 13 patients (30%) continued treatment and 30 patients (70%) had discontinued treatment because of disease progression (BRAFi, *n* = 12; BRAFi+MEKi, *n* = 8; BRAFi+Anti-PD-1 antibody, *n* = 6) or AEs (BRAFi+Anti-PD-1 antibody, *n* = 4). Almost all patients in the three groups experienced at least one AE (Table 2). Grade 3 or 4 treatment-related AEs were reported in four of 12 (33%), three of 12 (25%), and 11 of 19 (57.9%) patients in the vemurafenib, BRAFi+MEKi, and vemurafenib+anti-PD-1 antibody groups, respectively.

**Table 2:** Adverse events of three regimes.

***N* (%)**	**BRAFi**		**BRAFi+MEKi**		**BRAFi+PD-1 antibody**	
	***N*** **= 12**		***N*** **= 12**		***N*** **= 19**	
**Grade**	Any*	**III-IV**	Any*	**III-IV**	Any*	**III-IV**
Any adverse event	12 (100)	4 (33.3)	12 (100)	3 (25.0)	19 (100)	11 (57.9)
**Dermatological events**						
Rash	10 (83.3)	2 (16.7)	8 (66.7)	1 (8.3)	16 (84.2)	7 (36.8)
Palmo–plantar erythrodysesthesia	10 (83.3)	0	2 (16.7)	0	8 (42.1)	2 (10.5)
Alopecia	7 (58.3)	0	0	0	6 (31.6)	0
Pruritus	6 (50.0)	2 (16.7)	6 (50.0)	1 (8.3)	12 (63.2)	3 (15.8)
Photosensitivity reaction	3 (25.0)	0	0	0	6 (31.6)	0
Keratocanthoma	2 (16.7)	0	0	0	8 (42.1)	0
**Musculoskeletal events**						
Arthralgia	7 (58.3)	2 (16.7)	4 (33.3)	1 (8.3)	16 (84.2)	4 (21.1)
Myalgia	5 (41.7)	0	3 (23.1)	0	14 (53.8)	1 (3.8)
**Gastrointestinal events**						
Diarrhea	3 (25.0)	0	1 (8.3)	0	4 (21.1)	0
Nausea	3 (25.0)	0	3 (25.0)	0	3 (15.8)	0
Vomiting	3 (25.0)	0	2 (16.7)	0	3 (15.8)	0
**General disorders**						
Fatigue	4 (33.3)	0	3 (25.0)	0	9 (47.4)	0
Pyrexia	2 (16.7)	0	7 (58.3)	2 (16.7)	4 (21.1)	1 (5.3)
Headache	1 (8.3)	0	2 (16.7)	0	3 (15.8)	0
Dizziness	1 (8.3)	0	2 (16.7)	0	3 (15.8)	0
**Investigations/laboratory examinations**						
Increased alanine aminotransferase level	3 (25.0)	1 (8.3)	4 (33.3)	1 (8.3)	7 (36.8)	0
Increased aspartate aminotransferase level	1 (8.3)	1 (8.3)	3 (25.0)	1 (8.3)	4 (21.1)	0
Increased bilirubin	0	0	0	0	6 (31.6)	0
Increased blood creatinine	1 (8.3)	0	3 (25.0)	0	4 (21.1)	0
Hyperglycemia	3 (25.0)	0	6 (50.0)	0	5 (26.3)	0
**Pulmonary events**						
Cough	0	0	2 (16.7)	0	1 (5.3)	0
Pneumonia	0	0	0	0	1 (5.3)	0
**Endocrine dyscrasia**						
Hypothyroidism	0	0	0	0	3 (15.8)	0
Hyperthyroidism	0	0	0	0	1 (5.3)	0

Any*, any grade; AE, adverse event; ALT, alanine aminotransferase; AST, aspartate aminotransferase; CTC, common toxicity criteria; BRAFi, BRAF inhibitor; MEKi, MEK inhibitor.

Similar to the pivotal clinical trials in Caucasian patients (5, 23) compared with the vemurafenib group, the frequencies of cutaneous toxicity [rash (66.7 vs. 83.3%), palmoplantar erythrodysesthesia (16.7 vs. 83.3%), keratoacanthoma (0 vs. 16.7%)], and arthralgia (33.3 vs. 58.3%) were lower in patients in the BRAFi group combined with the MEKi group. This mainly resulted from paradoxical activation of the MAPK pathway due to BRAFi monotherapy (24, 25). Meanwhile, photosensitivity (25.0 vs. 0%) was more common in the vemurafenib group, which is commonly described in patients using vemurafenib (5), and did not occur with dabrafenib or trametinib treatment (26). MEKi-specific AEs included serious pyrexia (16.7%), which was more common in the BRAFi+MEKi group. In both BRAFi and BRAFi+MEKi groups, the most common grade ≥3 AEs included rash, pruritus, arthralgia, and increased aminotransferase levels, except for pyrexia (16.7 vs. 0%), which was similar in both groups. In the BRAFi group, four patients (33.3%) modified the dose of vemurafenib to 720 mg twice daily due to grade 3 AEs [arthralgia, *n* = 2; rash, *n* = 1; increased aminotransferase levels (ALT and AST), *n* = 1]. None of the patients discontinued therapy due to AEs. In the BRAFi+MEKi group, no AEs leading to dose modification or treatment discontinuation were reported because most treatment-related AEs were of grades 1–2 and could be alleviated with medications (antiallergic drugs, steroids, or hepatoprotective drugs). Only one patient experienced a grade 3 AE involving increased levels of both ALT and AST and interrupted D+T. The patient received appropriate treatment with hepatoprotective drugs to restore ALT and AST levels (Supplementary Table 1).

In the vemurafenib+anti-PD-1 antibody group, grade 3–4 AEs (57.9%) included rash (36.8%), arthralgia (21.1%), pruritus (15.8%), palmoplantar erythrodysesthesia (10.5%), and myalgia (3.8%). These AEs usually were more severe after the addition of an anti-PD-1 antibody. Eight patients experienced grade 3 rash or palmoplantar erythrodysesthesia, which occurred before the addition of anti-PD-1 antibody in one patient and which occurred after the addition of anti-PD-1 antibody in the other seven patients. Musculoskeletal AEs occurred frequently, especially arthralgia (62.8%), which worsened with continued treatment, but could be alleviated by lowering the dose of BRAFi. Pneumonitis was reported in one patient but no action was taken. Signs of altered imaging on CT scans disappeared after 2 weeks. Hypothyroidism and hyperthyroidism were reported in three patients and one patient, respectively. Both events were treated with endocrine therapy and those patients ultimately continued with the regimen (vemurafenib+anti-PD-1 antibody). Eighteen patients (94.7%) modified the dose of vemurafenib. Of these, four patients (21.1%) were still unable to tolerate AEs (grade 3 of fatigue: *n* = 1; iritis: *n* = 1; grade 3 rash: *n* = 2) and had finally discontinued the combined regimen. Two patients (10.5%) required a dose reduction of vemurafenib both before (decreased 960–720 mg twice daily) and after (720 mg decreased to 480 mg twice daily) the addition of anti-PD-1 antibody. Before the addition of the anti-PD-1 antibody, five patients (26.3%) required a reduction in the dose of vemurafenib to 480 mg two times daily due to AEs. The remaining 11 patients (57.9%) reduced the dose of vemurafenib after the addition of anti-PD-1 antibody, and four of these 11 patients eventually discontinued the combined therapy. Ten patients modified the vemurafenib dosage to 480 mg two times daily, two patients modified their dosage to 720 mg two times daily, and two patients modified vemurafenib treatment to 240 mg two times daily. Ten patients required a dose reduction of vemurafenib due to rash or arthralgia. We observed that two patients who received a dose reduction of vemurafenib to 240 mg achieved a complete response (CR) and stable disease (SD), respectively, and both CR and SD were maintained up to the last follow-up, which indicated that the combination of low-dose vemurafenib and anti-PD-1 antibody could also benefit patients. There was no significant difference in the ORR between the three groups with different doses of vemurafenib (ORR: 100% of 720 mg; 70% of 420 mg; 50% of 240 mg). However, it is possible that no significant differences were observed due to sample size limitations.

We defined trHE as the increase of either ALT, AST, or bilirubin in this study. Treatment with hepatoprotective drugs (e.g., polyene phosphatidylcholine, compound glycyrrhizin, and reduced glutathione) could decrease the aminotransferase levels and reduce the bilirubin levels (e.g., ademetionine 1,4-butanedisulfonate). All patients with trHE only presented laboratory abnormalities and had no clinical symptoms. Overall, 18 patients (41.9%) developed trHE (Supplementary Table 1). In the BRAFi group, trHE was reported in three patients and one patient experienced a grade 3 increase in both ALT and AST levels. In the BRAFi+MEKi group, trHE was reported in four patients and one patient experienced grade 3 increase of both ALT and AST levels. Increased bilirubin was not reported in either the BRAFi or BRAFi+MEKi groups. Two patients developed grade 3 trHE (elevated AST and ALT levels), which was successfully resolved by interruption of therapy and treatment with hepatoprotective drugs; the patients subsequently continued their therapy without any trHE relapse. In the BRAFi+anti-PD-1 antibody group, four patients (21.1%) experienced a grade 1 increase in bilirubin levels 1 month after the addition of the anti-PD-1 antibody. However, the levels returned to normal after ~4 weeks following treatment with ademetionine and polyene phosphatidylcholine. Five patients (26.3%) developed an increase either of ALT or AST levels within 4 weeks after the addition of the anti-PD-1 antibody, which resolved after 0.5–4 weeks with hepatoprotective drugs (*n* = 4) or recovered spontaneously (*n* = 1). Similarly, the two remaining patients (10.5%) showed a concomitant increase of bilirubin and aminotransferases (ALT and AST) 3 weeks after the addition of the anti-PD-1 antibody to the treatment, although they resumed the regime after treatment with hepatoprotective drugs.

In this study, four of 43 patients received a third-line therapy (BRAFi: *n* = 2, BRAFi+MEKi: *n* = 1, vemurafenib+pembrolizumab: *n* = 1), and only one patient (BRAFi+MEKi) received a fourth-line treatment. Nineteen of 43 patients received a second-line therapy (BRAFi: *n* = 6, BRAFi+MEKi: *n* = 6, vemurafenib+pembrolizumab: *n* = 7) (Table 1). For patients receiving a second-line treatment or more, their primary therapies included only chemotherapy, such as taxinol combined with cis-platinum complexes (DDP), dacarbazine (DTIC) combined with DDP, or temozolomide (TMZ) combined with paraplatin. The common clinical AEs associated with these chemotherapeutic drugs included short-term toxicity, which were relieved before the start of this study. Similarly, the efficacy of these chemotherapeutic agents could be observed in short term (2–3 months), which is in contrast to similar delayed effects observed with ICIs.

### AEs and Clinical Response Analysis

**Figure 1:**
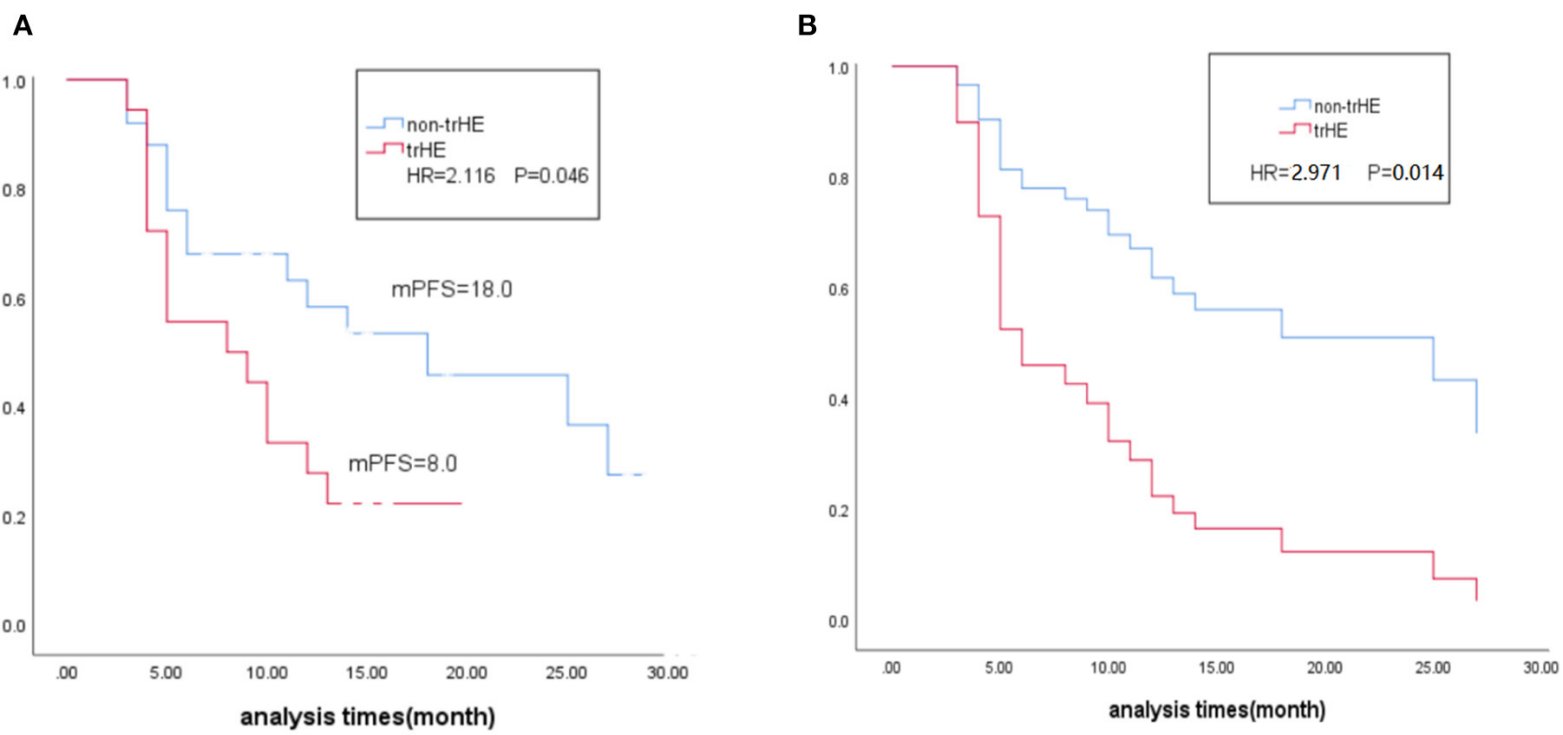
**(A)** Kaplane-Meier survival estimates in relation to occurrence of trHE. **(B)** Multivariate Cox regression estimates in relation to occurrence of trHE, corrected for baseline LDH level, medication regimens and liver metastases. trHE, treatment-related hepatotoxicity defined as the increase of either ALT, AST, or bilirubin levels; mPES, median progression-free survival; HR, hazard ratio; LDH, lactate dehydrogenase.

**Figure 2:**
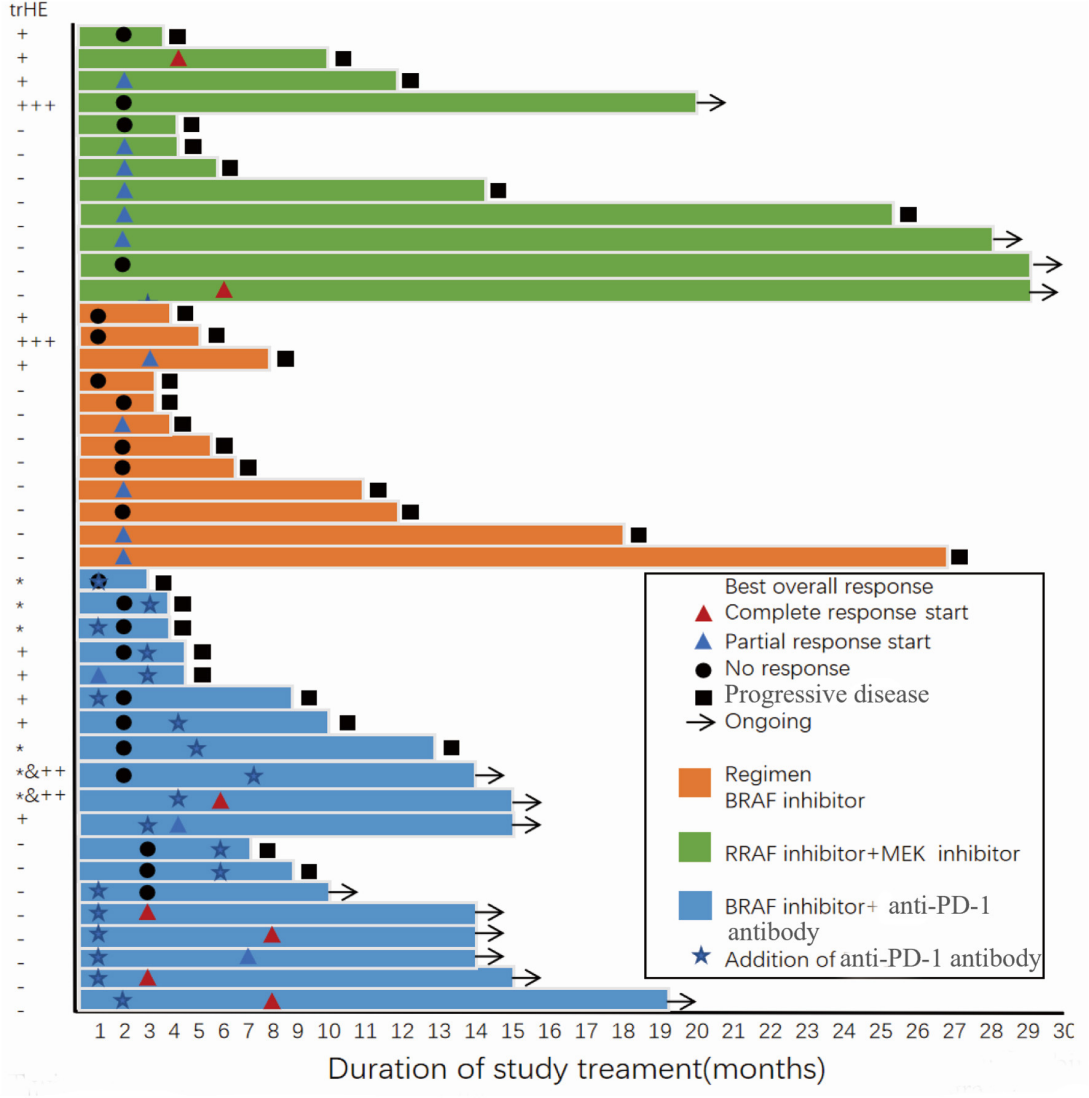
Progression-free survival and best overall response of all patients in the two groups with or without treatment-related hepatotoxicity. trHE, treatment-related hepatotoxicity defined as the increase of either ALT, AST, or bilirubin levels; +, grade 1 increase of either ALT or AST level; + + +, grade 3 increase of either ALT or AST level; *, grade 1 increase of bilirubin; ++&*, concomitant increase of bilirubin (grade 1) and either ALT or AST (grade 2).

After 22 AEs were screened by the Kaplan-Meier survival analysis, only trHE showed a significant correlation with PFS. Univariate analyses revealed that PS, LDH level, liver metastases, treatment regimens, as well as trHE, were significant prognostic indicators for PFS (Table 3). The results of the multivariate analysis indicated that TrHE retained its significance as a predictive factor, whereas PS, LDH levels, liver metastases, and treatment regimens provided no significant prognostic value for PFS (Table 4). The median PFS of patients with trHE was 8 months compared with 18 months for the remaining patients [*p* = 0.046, hazard ratio (HR) = 2.116, Cox regression analysis; Figure 1A]. Moreover, this association was independent of the baseline LDH level, medication regimens, PS, or the presence of liver metastases (*p* = 0.014, HR = 2.971, Cox regression analysis; Figure 1B). The ORR of patients with trHE was significantly lower than in those without trHE (44.4 vs. 60.0%, *p* = 0.024), and we observed a similar trend in patients treated with BRAFi (33.3 vs. 44.4%, *p* = 0.110), BRAFi+MEKi (50 vs. 75%, *p* < 0.001), and BRAFi+anti-PD-1 antibody (45.5 vs. 62.5%, *p* = 0.016) (Table 5). In the BRAFi group, 0/3 (0%) patients experienced trHE and achieved a CR to therapy, 1/3 (33.3%) achieved a partial response (PR), and 2/3 (66.7%) had SD as per RECIST v1.1 criteria. In the BRAFi+MEKi group, 1/4 (25%) with trHE achieved a CR, 1/4 (25%) achieved a PR, and 2/4 (50%) had SD. In the group treated with BRAFi+anti-PD-1 antibody, patients developing trHE achieved lower CR than those without trHE (18.1 vs. 50.0%, *p* < 0.001). The PFS of all patients in the two groups, with or without treatment-related hepatotoxicity, are shown in Figure 2. According to Supplementary Table 2, there were no statistically significant differences in age, initial LDH level, number of diseases, receiving first-line treatment or not, or the presence of liver metastasis between patients experiencing hepatotoxicity and those without hepatotoxicity.

**Table 3:** Univariate analyses of prognostic factors for PFS in patients with malignant melanoma.

**Characteristics**	**mPFS (month)**	**Univariate analyses (*P*-value)**
**Age**		0.768
≤45 years	12	
>45 years	9	
**Gender**		0.627
Men	11	
Female	12	
**Performance status**		0.001
0	25	
1	6	
2	4	
**LDH**		0.002
≤ULN	14	
>ULN	5	
**Disease sites**		0.308
≤3	14	
>3	6	
**Liver metastases**		0.024
Yes	5	
No	14	
**CNS metastases**		0.107
Yes	5	
No	12	
**Regimen**		0.016
BRAFi	5	
BRAFi+MEKi	12	
BRAFi+anti-PD-1 antibody	Not reach	
**TrHE**		0.046
Yes	8	
No	18	

*LDH, lactate dehydrogenase; BRAFi, BRAF inhibitor; MEKi, MEK inhibitor; trHE, treatment-related hepatotoxicity defined as the increase of either ALT, AST or blood bilirubin levels*.

**Table 4:** Multivariate analyses of prognostic factors for PFS.

**Prognosticators**	***P*-value**
Performance status	0.371
LDH	0.107
Liver metastases	0.533
Regimen	0.166
TrHE	0.014

*LDH, lactate dehydrogenase; Regimen, including BRAFi, BRAFi+MEKi and BRAFi+anti-PD-1 antibody; trHE, treatment-related hepatotoxicity defined as the increase of either ALT, AST or bilirubin levels*.

**Table 5:** Efficacy outcomes.

**Outcome**	**trHE**	**Non-trHE**
All	*n* = 18	*n* = 25
Confirmed ORR, *n* (%) [95% CI]	8 (44.4) [19.02–69.87]	15 (60) [39.36–80.64]
CR	3 (16.7)	5 (20)
PR	5 (27.8)	10 (40)
SD	9 (50)	10 (40)
PD	1 (5.6)	0
DCR	17 (94.4)	25 (100)
Median PFS, months (95% CI)	8.0 (0–16.32)	18.0 (4.56–31.43)
BRAFi	*n* = 3	*n* = 9
ORR	1 (33.3)	4(44.4)
CR	0	0
PR	1 (33.3)	4 (44.4)
SD	2 (66.7)	5 (45.6)
DCR	3 (100)	9 (100)
BRAFi+MEKi	*n* = 4	*n* = 8
ORR	2 (50)	6 (75)
CR	1 (25)	1 (12.5)
PR	1 (25)	5 (62.5)
SD	2 (50)	2 (25)
DCR	4 (100)	8 (100)
BRAFi+anti-PD-1 antibody	*n* = 11	*n* = 8
ORR	5 (45.5)	5 (62.5)
CR	2 (18.1)	4 (50)
PR	3 (27.3)	1 (12.5)
SD	5 (45.5)	3 (37.5)
PD	1 (9.1)	0
DCR	9 (90.9)	8 (100)

*ORR, overall response rate; CI, confidence interval; CR, complete response; PR, partial response; SD, stable disease; PD, progressive disease; DCR, disease control rate; PFS, progression-free survival*.

## Discussion

Targeted therapy with MAPKi regimens has dramatically changed the landscape of treatment of *BRAF*-mutant metastatic melanoma. Currently, the combination of MAPKi and ICIs may be a potentially effective approach to treat malignant melanoma. However, the toxicity spectrum of these therapies in different ethnic groups may be variable and will impact the treatment efficacy. Therefore, a description of the real-world experience regarding the tolerability and safety of BRAFi and BRAFi-based combinations in Chinese patients is significant.

In this study, patients receiving BRAFi monotherapy (vemurafenib) generally developed a similar pattern of AEs compared with Caucasian patients, and the occurrence of AEs appeared to be proportional to the dose of the drug (27). Compared with a study of vemurafenib treatment in 3,219 Caucasian patients, the Chinese patients in this study experienced higher incidence of aminotransferase level increase (25 vs. 0%) and hyperglycemia (25 vs. 0%). The most common grade 3 or higher AEs were keratoacanthoma (8%) and squamous cell cancer (cuSCC) of the skin (8%) in 3,219 Caucasian patients, which differed from this study (2). In the present study, arthralgia (16.3%) and rash (8.3%) were the most common grade 3 AEs, and all keratoacanthoma were grade 1–2 (16.7%). CuSCC was not observed in this study, and a similar rate as in reported in this study was observed in a phase I/II study of vemurafenib in Japanese patients with melanoma (28). Compared with Caucasian patients, the frequency of AEs (100 vs. 90%) was higher in this study; however, the frequency of grade 3/4 AEs (33 vs. 37%) in this study was lower, and no grade 4 AEs were observed among the patients. Meanwhile, the vemurafenib group of this study reported a higher incidence of liver system-related AEs (aminotransferase level increase) than in Caucasians. Differences between Asian and Caucasian populations might potentially lead to associated differences in the incidence of AEs. Since plasma concentrations of vemurafenib were generally consistent between Asian and Caucasian patients (16), differences in AE could be attributed to differences in culture and lifestyle, such as duration of exposure to sunlight and diet, as well as to differences in genetic susceptibility. Finally, compared with Caucasian patients, Chinese patients demonstrated equivalent or even better tolerance of BRAFi monotherapy.

Most cutaneous side effects, especially rash, pruritus, and palmar-plantar dysesthesia, decreased in the combination therapy group (D+T) compared with the BRAFi monotherapy treatment group in this study. A review evaluating AEs in Caucasians receiving V+C, D+T, and E+B reported that the targeted combination regimen resulted in fewer skin toxicities and more gastrointestinal side effects, particularly vomiting and diarrhea, which were probably caused by the MEKi (29). This was consistent with our observations regarding skin toxicity, but differed from the gastrointestinal AEs observed in this study. Furthermore, compared to Caucasians in the COMBI-V clinical trial, the patients receiving D+T had higher any grade incidence of arthralgia (33.3 vs. 26.6%) and skin toxicity, especially rash (66.7 vs. 24%) and pruritus (50 vs. 10%), but less gastrointestinal AEs, including diarrhea (8.3 vs. 34%), nausea (25 vs. 36%), and vomiting (16.7 vs. 31%) (29, 30). There was little difference in the incidence of pyrexia (any grade) between the BARFi+MEKi group and the COMBI-V group (58 vs. 55%). In the BRAFi+MEKi group, the most common grade 3 AE was pyrexia (16.7%), while it was less frequent (4.6%) in the COMBI-V study. Moreover, grade 3 hypertension (15.8%) was more frequent in the COMBI-V study compared to the Chinese patients included in this study.

Recently, a clinical trial evaluating dabrafenib (300 mg twice daily), trametinib (2 mg once daily), and pembrolizumab (2 mg/kg every 3 weeks) was conducted in 60 patients with *BRAF V600E/K*-mutated metastatic melanoma. It was suggested that this triple-combined therapy might benefit a subset of Caucasian patients with manageable AEs (20). Compared with the triple-drug combination group, the BRAFi+anti-PD-1 antibody group had higher AEs of different grades (100 vs. 98.3%) and lower AEs of grades 3–4 (58 vs. 70%). The most common AEs in our BRAFi+anti-PD-1 antibody group were skin-related toxicities, with an incidence of up to 95%, and included rash (84.2%), pruritus (63.2%), and palmoplantar erythrodysesthesia (42.1%), followed by arthralgia (84.2%), fatigue (47.4%), and increased ALT or AST (26.3%) levels. However, in the triple-combination therapy, pyrexia (80%) was the most frequent AE, followed by rash (41.7%), diarrhea (40%), and nausea (35%). Pyrexia in our BRAFi+anti-PD-1 antibody group was clearly less frequent compared to the triple-combined therapy (any grade: 21 vs. 80%, grade 3: 5 vs. 11.7%), which was probably was due to the absence of MEKi and sequential therapy in our study. Severe treatment-related AEs also differed between the two groups. In this study, the most common grade 3 AEs were rash (36.8%) and arthralgia (15.8%), compared to increased ALT or AST levels (15%) and pyrexia in the triple-combination therapy. In addition, six patients (31.6%) in our group experienced increased bilirubin levels, which were not reported in the triple-drug combination clinical trial. Most severe AEs in the BRAFi+anti-PD-1 antibody group could be alleviated by temporarily interrupting treatment and subsequently reducing the dose of vemurafenib; however, four patients (21%) were still unable to tolerate this treatment. Compared to this study, 25 Caucasians (41.7%) in the triple-combined therapy group discontinued treatment and six of them terminated the therapy due to grade 3–4 increased AST or ALT levels. Compared with the triple-drug combination therapy, hepatotoxicity in the BRAFi+anti-PD-1 antibody group was mostly grade 1–2, which could be alleviated by symptomatic liver protection treatment. The reasons for interrupting the treatment were severe skin or arthralgia toxicity 1 or 2 weeks after starting the therapy. Overall, 94.7% of patients in the BRAFi+anti-PD-1 antibody group underwent dose reduction of vemurafenib, which might have contributed to the reduced incidence of subsequent severe hepatotoxicity.

From the above data, it may be concluded that the tolerance level of the BRAFi +PD-1 antibody regimen by Chinese patients was acceptable. A similar conclusion could be drawn from another phase I triple-drug trial on 15 Caucasians when compared with this study (21).

We defined trHE as an increase of either ALT, AST, or bilirubin. However, distinguishing these laboratory indicators based on either the treatment induced or liver metastasis is discussed below. First, 18 patients had normal ALT, AST, and bilirubin levels before starting treatment with BRAFi or BRAFi-based combinations and did not receive any other medicines that potentially lead to liver injury. Further, trHE was reported after receiving treatment. trHE was relieved after receiving hepatoprotective drugs. Second, four patients had liver metastasis before experiencing trHE, and there was no evidence about the progression of their liver metastasis while experiencing trHE. The remaining patients did not experience any liver metastasis either before or after trHE as determined by imaging studies. In addition, only one patient had a history of hepatitis B, and the DNA load of the hepatitis B virus showed no significant enhancement. Finally, none of the patients experienced a secondary trHE in subsequent treatment. However, these phenomena might have resulted from our small sample size and short observation time.

Patients experiencing trHE had shorter PFS and lower ORR, which we speculated occurred for the following possible reasons. First, different prognosis may be influenced by the general condition of the patient, staging, liver involvement, basic liver metastasis, and treatment. Further analysis showed that there was no statistical difference between the two groups (with or without trHE) in terms of age, first-line treatment, LDH level, lesion number, and other basic status indicators (Supplementary Table 2). Second, glycyrrhizic acid, a hepatoprotective drug and the main ingredient of compound glycyrrhizin glucoside [stronger neo-minophagen C (SNMC)], has anti-inflammatory and anti-allergic effects and exhibits steroidal hormone-like properties (31). Thus, glycyrrhizic acid may be a potential inhibitor of the immune response. In this study, three patients (in each of the three groups) were treated with SNMC for trHE and all of them achieved SD. Due to the limited sample size, it was difficult to determine the positive or negative effect of SNMC. Finally, we questioned the impact of reducing vemurafenib or treatment termination on the prognosis of patients in the trHE group. However, after analysis, in the BRAFi group, we found that only one patient with trHE had received a reduced dose of vemurafenib. The best response of the patient was PR, and his PFS was 6 months, which reached the general PFS observed with BRAFi monotherapy. No other patient had terminated treatment. In the BRAFi+anti-PD-1 antibody group, trHE did not lead to discontinuation. Only one patient required a dose reduction of vemurafenib due to trHE. He had SD, which was maintained up to the follow-up deadline (PFS = 14 months).

The present study has some limitations, such as treatment selection bias due to the retrospective nature of the study, the single-center analysis, and the small study sample. Essentially, the underlying mechanism of hepatotoxicity induced by BRAFi and BRAFi-based combined regimens and its relationship with treatment outcomes need to be further explored.

In conclusion, in this study, treatment-related AEs in the Chinese population receiving BRAFi and BRAFi-based regimens were generally consistent with those reported in Caucasians, although the occurrence of grade 3 AEs was lower in Chinese patients. The trHE in patients receiving BRAFi and BRAFi-based regimens may indicate a poor treatment-related prognosis.

## Data Availability Statement

The original contributions presented in the study are included in the article/Supplementary Material, further inquiries can be directed to the corresponding author/s.

## Ethics Statement

The studies involving human participants were reviewed and approved by Ethics Committee of Sun Yat-sen University Cancer Center, Sun Yat-sen University. The patients/participants provided their written informed consent to participate in this study.

## Author Contributions

X-sZ: study design and concepts. XL, J-jL, D-sW, X-zW, D-dL, YD, J-hW, and HJ: data acquisition. XL and J-jL: quality control of data, algorithms, data analysis, and interpretation. XL: statistical analysis, manuscript preparation, and manuscript editing. All authors read and approved the final manuscript.

## Conflict of Interest

The authors declare that the research was conducted in the absence of any commercial or financial relationships that could be construed as a potential conflict of interest.
