# Supplementary material for: Safety and Tolerability of BRAF Inhibitor and BRAF Inhibitor-Based Combination Therapy in Chinese Patients With Advanced Melanoma: A Real World Study
Source: Front Oncol. 2021 Apr 1;11:582676. doi: 10.3389/fonc.2021.582676 (PMC8047100; doi:10.3389/fonc.2021.582676)
Supplement: Supplementary file 1 [file Table_1.DOCX]

Supplementary Table 1 Drug treatment in patients with treatment-related hepatotoxicity.

|  | trHE | Toxicity  grade | Duration | Related to  treatment (Y/N) | Action taken | Take medicine | Outcome |
| --- | --- | --- | --- | --- | --- | --- | --- |
| BRAFi group | | | | | | | |
| 1 | increased ALT | 3 | 5 weeks | Y (V) | Reduced (V) | PPC,prednisone,GGI | Resolved |
|  | increased AST | 3 | 5 weeks | Y (V) | Reduced (V) |  | Resolved |
| 2 | increased ALT | 1 | 3 weeks | Y (V) | None | PPC | Resolved |
| 3 | increased ALT | 1 | 4 weeks | Y (V) | None | None | Resolved |
| BRAFi+MEKi group | | | | | | | |
| 1 | increased ALT | 3 | 12 weeks | Y (D, T) | Interrupted (D, T) | PPC, GSH, GGT | Resolved |
|  | increased AST | 3 | 12 weeks | Y (D, T) | Interrupted (D, T) |  | Resolved |
| 2,3 | increased ALT | 1 | 3 weeks | Y (D, T) | None | PPC | Resolved |
|  | increased AST | 1 | 3 weeks | Y (D, T) | None |  | Resolved |
| 4 | increased ALT | 1 | 3 weeks | Y (D, T) | None | None | Resolved |
| BRAFi+anti-PD-1 antibody group | | | | | | | |
| 1 | increased bilirubin | 1 | 4 weeks | Y (pembro, V) | None | T , PPC | Resolved |
| 2 | increased bilirubin | 1 | 3.5weeks | Y (pembro, V) | None | T , PPC | Resolved |
| 3 | increased bilirubin | 1 | 4weeks | Y (pembro, V) | None | T , PPC | Resolved |
| 4 | increased bilirubin | 1 | 4 weeks | Y (pembro, V) | None | T , PPC | Resolved |
| 5 | increased ALT | 1 | 0.5 weeks | Y (pembro, V) | None | PPC | Resolved |
| 6 | increased ALT | 1 | 2weeks | Y (pembro, V) | None | PPC | Resolved |
| 7 | increased ALT | 1 | 3weeks | Y (pembro, V) | None | None | Resolved |
| 8 | increased ALT | 1 | 3 weeks | Y (pembro, V) | None | PPC | Resolved |
|  | increased AST | 1 | 3 weeks | Y (pembro, V) | None | PPC | Resolved |
| 9 | increased ALT | 1 | 2.5weeks | Y (pembro, V) | None | PPC | Resolved |
|  | increased AST | 1 | 2.5weeks | Y (pembro, V) | None | PPC | Resolved |
| 10 | increased bilirubin | 1 | 8 weeks | Y (pembro, V) | Reduced (V) | T, PPC, GGT | Resolved |
|  | increased ALT | 2 | 6weeks | Y (pembro, V) | Reduced (V) | T, PPC, GGT | Resolved |
|  | increased AST | 1 | 3weeks | Y (pembro, V) | Reduced (V) | T, PPC, GGT | Resolved |
| 11 | increased bilirubin | 1 | 4 weeks | Y (pembro, V) | Interrupted (V) | T, PPC, GGT | Resolved |
|  | increased ALT | 2 | 3weeks | Y (pembro, V) | Interrupted (V) | T, PPC, GGT | Resolved |
|  | increased AST | 1 | 2.5weeks | Y (pembro, V) | Interrupted (V) | T, PPC, GGT | Resolved |

N no use drug; PPC polyene phosphatidyl choline; GGI compound glycyrrhizin injection; GSH reduced glutathione; GGT compound glycyrrhizin tablets; T ademetionine 1,4-butanedisulfonate

BRAFi, BRAF inhibitor; MEKi, MEK inhibitor

Supplementary table 2 Patient and disease characteristics according to the occurrence of hepatotoxicity

|  | Age | Normal LDH | Disease site | First line | Liver metastases |  |  |
| --- | --- | --- | --- | --- | --- | --- | --- |
|  |  | level |  |  |  |  |  |
|  | (median) | (%) | (median) | (%) | (%) |  |  |
| trHE(n=18) | 45.5y | 57.9 | 4 | 36.8 | 57.9 |  |  |
| non-trHE(n=25) | 46.4y | 67.9 | 3 | 27.3 | 64.3 |  |  |
| P vulue | 0.601 | 0.462 | 0.277 | 0.251 | 0.444 |  |  |
